# 

**Published:** 1911-03-11

**Authors:** 


					J&35 tHHuod. Ninth Edition, Rewritten and Enlarged, 9s. net.
PHARMACY, MATERIA MEDICA & THERAPEUTICS.
Being a Ooairjuaitary on the B.P. and its Drugs, with their Preparations,
and on all Uic new Non-official Remedies in use, with Chapters on the
different processes used in Dispensing, Prescription-writing, Latin
Glossary. <fcc By Sip W. WHITLA, M.D., LL.D., Professor, Materia
Medica, Quaaa'H University, Belfast, and Senior Physician, Boyal Victoria
Hospital.
By the sine iuthor. New Work. 1,900 pages. Orown8vo.25s.net.
PRACTICE OF MEDICINE.
Containing i:t {wo Handy Volumes a complete and independent Article on
the Etiology. Hvuiptoms, and Diagnosis of every Medical Disease; arranged
ii Dictionary Form for Practitioners and Students.
I,oud.rt CAjttritRK, Ttndai.t. & Oox, 8 Henrietta Street, W.O.
JOTTINCS.
On Tuesday, June 13, the Countess of Dalhousie will
open, at 2.30 p.m., a three-days' sals of work at the Royal
Hospital for Incurables, Putney, and the Board has de-
c ded to hold a garden party at tha Hospital on Wednes-
day, July 5.
i. he Annual General Meeting cf the Poplar Hospital for
Accidents, Poplar, E., will be held at the Hospital on
Tuesday, March 21, 1911, at 4 o'clock p.m.
Mn. and Mrs. Fewla33 Llewellyn and their friends
are giving an entertainment to the patients of the Royal
.Hospital for Incurable.!, Putney, on Wednesday. March 29.
Mr. Nigel Effingham, the well-known humorist, is also
paying a vkit to this Home on the Hill at an early date.
Mr. Neville Priestley, of " Roadside," West Hill,
and Mr. R. S. St. Quintin, of " Duart," West Hill,
Putney, have recently been unanimously elected members
of the Board of Management cf the Royal Hospital for
Incurables, Putney, which is the largest and most historic
institution of it.3 special character in Great Britain.
The annual meeting of Governor,i and Subscribers of
Queen Charlotte's Lying-in Hospital will be held at the
Hospital on Tuesday, February 28, at 3 p.m. precisely, when
the following will be the agenda : (1) To receive the annual
report; (2) to receive tho audited accounts and balance-
sheets ; (3) to elect Governors; (4) to elect members of
Committee of Management; and (5) to elect auditors for
the ensuing year.
The Honourable Harry Lawson, M.P., will preside
at the next Fcctival Dinner of the Royal Hospital for
Incurables, Putney, which will be held at the Grocers'
Hall, Princes Street, E.C., on Thursday, May 4. The
next half-yearly election will be held at the Cannon Street
Hotel, on May 24, when thirty candidates will be elected.
Mr. Herbert John Allcroft, F.R.G.S., will be in the chair.
The annual general meeting of the University College
Hospital, Gower Street, W.C., will be held on Thursday,
March 16, in the Board Room of the Hospital, at 3.30 p.m.
precisely. The agenda is as follows : The election of a
president, vice-presidents, treasurer, of twelve members of
the general committee, and of a public accountant, and
the consideration of the reports of the condition and
prospects of the Hospital and Medical School respectively.
The Sheffield Corporation have decided to set aside
temporarily, during the present epidemic of measles, a
certain amount of accommodation in the city hospitals, such
to be reserved solely for such cases as cannot be treated
adequately at home. Cases already under the care of the
Guardians will not be admitted, and a circular to this effect,
has been sent by the Medical Officer of Health, Dr. Scurfield,
to the medical men in the district. The beds have been set
aside from February 20 onwards.
The eighty-third Annual Court of the Royal Free Hos-
pital, Gray's Inn Road, W.C., will be held at the Hospital
on Wednesday, March 15, when the chair will be taken, at
3.30 p.m., by H.R.H. Princess Christian of Schleswig-
Holstein, President of the Hospital. The annual reports
and accounts will be presented, and the Governors and
their friends invited to inspect the wards and the various
departments of the Hospital during the afternoon. Tea
will be served in the Board Room at the close of the
proceedings.
The proprietors of Jeyes' Fluid have had the honour of
receiving the only warrant of appointment for disinfectants
to her Gracious Majesty Queen Alexandra. Messrs. Jeyes
also hold the royal warrant to his Majesty King George Y.
Gifts and Bequests.
A second anonymous donation of ?50 from "An Old
Gravesend Tradesman " has been paid to the credit of the
funds of the Gravesend Hospital.
The Rev. Henry Sale, of the Rectory, Winteringham,
Iiincs., left ?100 each to the Church of England Waifs
and Strays Society, of Old Town Hall, Kennington, S.E.,
.and the Brompton Hospital for Cancer.
Mr. William Daly, of Dunsandle, Athenry, Co. Gal-
-way, bequeathed ?300 each to the Royal National Hospi-
tal for Consumption for Ireland at Newcastle, Co." Wick-
low, and St. Vincent's Hospital, Stephen's Green, Dublin ;
?200 to the Royal Victoria Eye and Ear Hospital, Dublin,
tin each case to found and endow a " Dunsandle " bed.
The late M. Auguste Loutreuil, one of the " Railway
Kings " of France, who made a large fortune in Rus sian
xailway enterprises and metallurgical industries, has bene-
fited French Science by testamentary dispositions amount-
ing to more than seven millions of francs (over ?280,000).
He bequeathed 100,000 francs to the Pasteur Institute, a
million francs to the Caisse des Recherches Scientifiques,
.two and a half millions to the University of Paris, and three
?and a half millions to the " Academic des Sciences." During
.his residence in Moscow he had been a generous supporter
of various charitable institutions.
Mr. John Roberts, of Kimberley Road, Sketty Cockett,
Glam., bequeathed, subject to a life interest, ?500 to the
Charing Cross Hospital, in recognition of the kindness re-
ceived by his late wife when she was an out-patient there,
and ?500 each to the Swansea Hospital and the Bristol
General Hospital.
At the twenty-fourth annual meeting of the Leeds Work-
people's Hospital Fund, held at the Town Hall, Leeds,
on February 22, with Mr. F. R. Spark, founder of the
Fund, as president, the general secretary read the report
and balance-sheet, which were in. every way satisfactory.
The popularity of the Fund among the workpeople of the
city increases every year. For the first time in its history
the amounts collected from large and small work places
within the city exceed ?10,000. The money has been
distributed as follows: General Infirmary, ?5,250;
Leeds Public Dispensary, ?800; Hospital for Women and
Children, ?800; Tuberculosis Association, ?315; Leeds
District Nursing Association, ?250; Leeds Maternity Hos-
pital, ?125; Bramley District Nursing Association, ?50;
Stanninglcy District Nursing Association, ?50; Leeds
Homoeopathic Dispensary, ?15; leaving ?1,134 13s. 9d.
to be carried forward to next vear's account.
BUILDINGS CONTEMPLATED AND
IN PROGRESS.
Rugeley.?Tenders are invited from builders for the
erection of two new wards, operating room, etc., at the
District Hospital, Rugeley. Mr. W. E. Rogers is the
architect.
Rio de Janeiro.?The Chamber of Deputies of Brazil
contemplate the erection of a Faculty of Medicine with a
clinical hospital in Rio de Janeiro, and invite architects to
submit schemes.
Rochdale.?Active steps are being taken to materialise
the extension scheme of Rochdale Infirmary. More than
?10,000 has been promised towards a scheme of twenty-
four beds, together with operating theatre and its
adjuncts, which is estimated to cost ?20,000.
Swansea.?Close on ?30,000 is the sum aimed at by
the Management Board of Swansea Hospital for the
hospital extension. The extension will provide seventy-
six new beds, besides four for casualties, as well as an
up-to-date casualty department and new dispensary.
Several munificent contributions have already been made.
Coventry.?For the erection of a nurses' home in con-
nection with the Workhouse Infirmary, the tender of ?3,723
by Mr. Lord, of Coventry, has been accepted. T. F. Tick-
ner, F.R.I.B.A., is the architect.
Ei'ping (Essex).?For the erection of an infirmary,
master's house, and contingent works, Messrs. Clark and
Sons' estimate of ?5,998 has been accepted.
Eastleigh and Bishopstoke.?Amended plans fcr the
Isolation Hospital have been submitted and approved by
the Urban District Council, and are being forwarded to the
Local Government Board for their preliminary approval.
THE WEEK'S NOVELTIES.
FAIR'S BEETLE PASTE is a simple and efficient
weapon against cockroaches, and as it is not touched by
animals, it is a safe remedy to employ In the household.
A tin should be available for every dispensary or surgery,
for in these it is often very difficult to exterminate these
insect pests. Fair's Paste may be relied upon to clear the
premises of black beetles with the least possible delay.
MEDICAL APPOINTMENTS, ETC.
Mr. Melville M. Adams, M.R.C.S., L.R.C.P. (Guy's),
has been appointed to succeed Mr. S. W. Milner as house
surgeon to the Gravesend Hospital.
Dr. Frank Keane, M.A., M.D., of Dr. Steevens' Hos-
pital, Dublin, has been appointed Assistant House Surgeon
to the West Bromwich District Hospital.
Mr. H. Tyrrell Gray, M.A., M.C.(Cantab),
F.R.C.S.Eng., has been appointed surgeon to out-patients
at the Hampstead General and North-West London
Hospitals.
APPOINTMENTS VACANT.
Full particulars of these vacancies can be obtained nn
reference to our advertisement columns :?
Mission Hospital, Nazareth, Palestine.?House
Surgeon.
Royal Free Hospital.?Senior Resident Medical Officer.
Tower Hamlets Dispensary.?Resident Medical Officer.
Wolverhamfton Genebal Hospital.?House Surgeon,
Resident Surgical Officer.
The latest addition to the fleet of the Yeoward Line, of
Liverpool, was launched on Thursday from the yard of the
Caledon Shipbuilding and Engineering Company, Limited,
Dundee, in the presence of a goodly gathering of visitors.
The steamer, which has been built to Lloyd's highest class
and Board of Trade requirements, is intended for service
in the owners' line running between Liverpool, Lisbon,
Madeira, and the Canary Islands, and has been specially
designed to give passengers the utmost amount of pleasure
and comfort, for which the steamers of this lin^ are so well
known. She is 290 ft. in length, 41 ft. 6 in. beam, and de-
signed to carry about 3,000 tons d.w., in addition to about
100 first-class passengers, whilst the vessel is also specially
adapted for the transport of bananas from the Canaries.
She hajs been christened "Andorinha," and, we under-
stand, is to be fitted with Marconi's wireless telegraphy.
Books for Review.
Publishers are particularly requested to send
advance proofs of any new books of importance,
whenever possible, as the Editor has made arrange-
ments to publish immediate reviews, and on a new
plan.
Correspondence.
Correspondence on all subjects is invited, but no
.communication can be entertained i? the name and
address of the correspondent are not given as a
guarantee of good faith, but not necessarily for pub-
lication. All correspondents should write on one
side of the paper only.
BUSINESS NOTICES.
Annual Subscriptions.
The rate of annual subscriptions to The
Hospital, either direct from the Office or through
agents, is: ?
For the United Kingdom   15e. a year.
For the Colonies and Abroad  17s. ,,
For the United Kingdom (with Insurance
Policy)    ?1 ?
inclusive of postage in each case.
Letters relating to the Publishing, Sale and Advertisement
Departments must be addressed to the Manager (not to the
Editor) :?The Manager. The Hospital Building, 28 and
29 Southampton Street, Strand, London, W.C.

				

